# Deep 2-photon imaging and artifact-free optogenetics through transparent graphene microelectrode arrays

**DOI:** 10.1038/s41467-018-04457-5

**Published:** 2018-05-23

**Authors:** Martin Thunemann, Yichen Lu, Xin Liu, Kıvılcım Kılıç, Michèle Desjardins, Matthieu Vandenberghe, Sanaz Sadegh, Payam A. Saisan, Qun Cheng, Kimberly L. Weldy, Hongming Lyu, Srdjan Djurovic, Ole A. Andreassen, Anders M. Dale, Anna Devor, Duygu Kuzum

**Affiliations:** 1 Department of Radiology, UCSD, La Jolla, CA 92093 USA; 2 Department of Electrical and Computer Engineering, UCSD, La Jolla, CA 92093 USA; 3 Department of Neurosciences, UCSD, La Jolla, CA 92093 USA; 4 NORMENT - KG Jebsen Centre for Psychosis Research, Division of Mental Health and Addiction, Oslo University Hospital and University of Oslo, 0407 Oslo, Norway; 5 Department of Medical Genetics, Oslo University Hospital, 0407 Oslo, Norway; 6 NORMENT, KG Jebsen Centre for Psychosis Research, Department of Clinical Science, University of Bergen, 5020 Bergen, Norway; 7 Martinos Center for Biomedical Imaging, MGH, Harvard Medical School, Charlestown, MA 02129 USA

## Abstract

Recent advances in optical technologies such as multi-photon microscopy and optogenetics have revolutionized our ability to record and manipulate neuronal activity. Combining optical techniques with electrical recordings is of critical importance to connect the large body of neuroscience knowledge obtained from animal models to human studies mainly relying on electrophysiological recordings of brain-scale activity. However, integration of optical modalities with electrical recordings is challenging due to generation of light-induced artifacts. Here we report a transparent graphene microelectrode technology that eliminates light-induced artifacts to enable crosstalk-free integration of 2-photon microscopy, optogenetic stimulation, and cortical recordings in the same in vivo experiment. We achieve fabrication of crack- and residue-free graphene electrode surfaces yielding high optical transmittance for 2-photon imaging down to ~ 1 mm below the cortical surface. Transparent graphene microelectrode technology offers a practical pathway to investigate neuronal activity over multiple spatial scales extending from single neurons to large neuronal populations.

## Abstract (web summary)

Optical imaging and manipulation technologies cannot be easily integrated with electrical recordings due to generation of light-induced artifacts. Here the authors report the optimization of transparent graphene microelectrode fabrication to achieve artifact-free electrical recordings along with deep 2-photon imaging in vivo.

## Introduction

Multimodal integration of sensing and manipulation technologies allows comprehensive investigation of brain function across spatiotemporal scales^1^. Multi-photon imaging has enabled cellular-resolution imaging of neural populations in animal models, whereas optogenetics (OGs) has been widely employed for selective control of neural activity and casual manipulation of specific neural circuits. High temporal resolution of electrophysiological recordings is critical to complement optical techniques for investigating fast dynamics of neural activity toward understanding functions of neural circuits. Furthermore, vast majority of early neuroscience research and clinical human studies rely on electrophysiological recordings of brain-scale activity. Combining optical techniques with electrical recordings can bridge the classical neuroscience knowledge obtained from animal models to human studies based on electrophysiological techniques. To that end, new tools allowing simultaneous measurements of multiple optical and electrical parameters are essential. Previous reports have shown proof-of-concept acquisition of local field potentials (LFPs) by graphene-based electrodes during simultaneous single-photon fluorescence imaging in vitro^2^, or OG photostimulation in vivo^3^. However, especially recordings during in vivo OG photostimulation have significantly suffered from light-induced artifacts, not showing any noticeable advantage over conventional platinum electrodes^3^. The problem of light-induced artifacts needs to be addressed, as it constitutes a major obstacle particularly for the adaptation of transparent graphene electrode technology in chronic in vivo studies.

Light-induced artifacts generated by photovoltaic (Becquerel effect) and photothermal effects appear as transients or oscillations in recordings and can interfere with LFP or spike recordings, depending on the frequency and duration of the light stimulus. The amplitudes of those artifacts are particularly higher for deep 2-photon imaging and OG stimulation^4–7^, due to increased laser power. Here, in the present study, we demonstrate that by careful design of key steps in the fabrication process for transparent graphene electrode, the light-induced artifact problem can be mitigated and virtually artifact-free LFP recordings can be achieved within operating light intensities. High optical transmittance of graphene supports simultaneous 2-photon imaging down to > 1 mm directly beneath the transparent microelectrodes. For the first time, we show that transparent graphene electrodes can be employed for crosstalk-free integration of three different modalities, 2-photon imaging, OGs, and electrical recordings of cortical potentials at the same time in the same experiment. We combine LFP recordings from the cortical surface, with simultaneous (i) 2-photon imaging of neuronal and vascular structure down to ~ 1 mm below the cortical surface, (ii) 2-photon imaging of neuronal calcium activity, (iii) single-photon OG photoactivation, (iv) 2-photon imaging of arteriolar vasodilation, and (v) large-scale optical imaging of hemodynamic responses. Crosstalk-free integration of various in vivo optical imaging and stimulation methods with graphene electrode recordings proves that transparent graphene technology is a versatile platform applicable to numerous different experimental settings. In cases where depth-resolved electrical recordings are not required, optically transparent graphene technology allows seamless integration with depth-resolved optical imaging and stimulation circumventing the need for inserting invasive probes into brain tissue.

## Results

### Elimination of crack formation and organic residues

In order to produce transparent graphene microelectrode arrays with high yield, uniformity and artifact-free recording capability, we improved three critical steps in the fabrication process: (1) graphene transfer, (2) photoresist removal from the graphene surface, and (3) graphene surface cleaning from organic residues. We first optimized the process of graphene transfer from growth to target substrate. This process is the most critical, yet most sensitive step of the fabrication. Previously used techniques, such as wet transfer^3^ and poly(methyl methacrylate) (PMMA) scaffold transfer^2^ are prone to formation of cracks on the monolayer graphene sheets resulting in low yield for large-area planar arrays. In addition, copper etching used in these processes leaves residues impeding the process of PMMA removal^8^. As a result, the graphene layers may lose their structural integrity during annealing. Therefore, we adopted an alternative “bubbling transfer” process^9^ for crack-free transfer of graphene sheets onto the polymer substrate (see Methods). To protect the graphene during photoresist stripping we used polymethylglutarimide (PMGI)-based bilayer lithography. Compared with other photoresists, PMGI leaves fewer residues on the graphene surface after resist removal. Photoresist removal is also critical to reduce crack formation in subsequent fabrication steps. To this end, we developed a four-step stripping/cleaning protocol for extensive cleaning of the graphene surface leading to a robust reduction of the average electrode impedance in the array (see Methods and Supplementary Fig. 1A, B). These removal and cleaning steps are also crucial to achieve a residue- and contamination-free graphene surface, which is essential to minimize light absorption and generation of light-induced artifacts by graphene electrodes (Supplementary Fig. 1C, D).

**Fig. 1:**
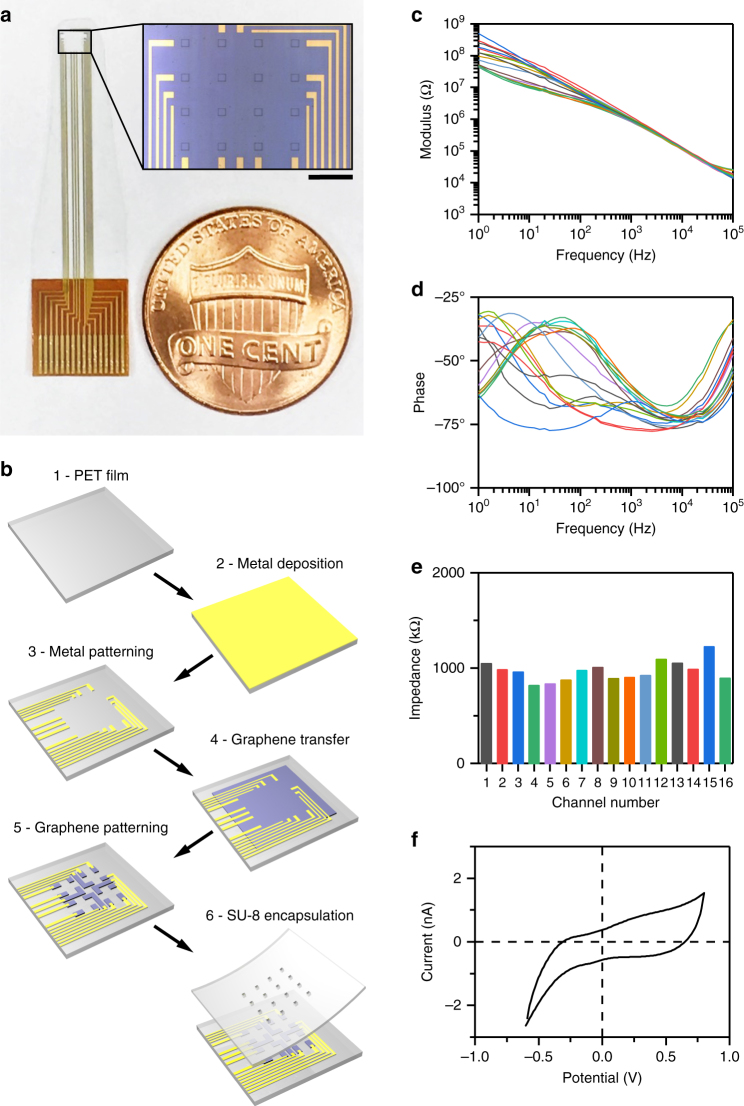
Graphene microelectrode array fabrication and electrochemical characterization. **a** Design of the graphene microelectrode array. Each electrode is a 100 µm×100 µm square and the spacing in-between two adjacent electrodes is 300 µm (edge-to-edge). Scale bar, 500 µm. **b** Fabrication of graphene microelectrode arrays. Step 1: clear 50 µm-thick PET film. Step 2: 10 nm Cr and 100 nm Au sputtered onto the PET film. Step 3: metal wires patterned with photolithography and wet-etching. Step 4: graphene transfer with bubbling method. Step 5: graphene contact pads patterned with photolithography and oxygen plasma-etching. Step 6: spin-coating of SU-8 and patterning of openings with lithography. **c**, **d** Electrochemical impedance spectroscopy of all 16 channels on an array. **e** Impedance at 1 kHz; channels have an average impedance of 963 kΩ. **f** Cyclic voltammetry of a representative channel shows no redox peaks

We fabricated 4-by-4 arrays of 100 µm × 100 µm square graphene electrodes separated by 300 µm (edge-to-edge) (Fig. 1a). Fabrication steps for building transparent graphene arrays are shown in Fig. 1b. The design included a gold array on the same substrate as the graphene arrays used as control sample. To fabricate transparent arrays, clear polyethylene terephthalate (PET, 50 µm thick) was chosen as substrate for its high optical transmittance (Supplementary Fig. 2). To provide mechanical support during fabrication, the PET film was placed on a 4-inch silicon wafer covered with polymethylsiloxane (PDMS) as adhesive layer. Layers of 10 nm chromium and 100 nm gold were deposited onto PET film. Metal wires were patterned with photolithography and wet-etching. Graphene was then transferred onto the designed area with the bubbling transfer method (see Methods). Graphene electrodes, wires, and contact pads were patterned with photolithography and oxygen plasma etching, and photoresist removal and cleaning of the graphene surface were performed as described in Methods. Finally, the whole array was encapsulated by an 8 µm-thick SU-8 layer with openings only at active electrode areas.

### In vitro characterization of graphene microarrays

Following the fabrication, we characterized each graphene microelectrode array in 0.01 M phosphate-buffered saline (PBS) with electrochemical impedance spectroscopy and cyclic voltammetry (CV) (Fig. 1c–f). Figure 1e shows a uniform impedance distribution across 16 channels at 1 kHz as a representative sample. Our improved fabrication process results in a uniform impedance distribution and high yield; 95–100% of the channels have impedances < 1.5 MΩ. Although high-impedance channels can still record neuronal activity, impedances < 1.5 MΩ are desirable to minimize noise and obtain high signal-to-noise ratio (SNR) recordings (Supplementary Fig. 3). CV measurements (Fig. 1f) exhibit no redox peaks, suggesting that the graphene/solution interface is capacitive. To test for sufficient flexibility of the graphene microelectrode array, we performed bending tests where the microarray was repeatedly bent to a radius of 5 mm without signs of device failure after 120 bending cycles (Supplementary Fig. 4). A radius of 5 mm is within the same range as the natural curvature of mouse cortex.

To examine to which extent the presence of the graphene microarray affects the resolution of images acquired with 2-photon laser scanning microscopy, we placed microarray devices in a phantom sample above mounting medium containing 1 µm fluorescent polystyrene beads at a low concentration (Supplementary Fig. 5). Compared with beads imaged beside the graphene microarray, a moderate (1.2- to 1.4-fold) increase in apparent bead size (full-width half-maximum (FWHM) within the imaging plane, i.e., across *X* and *Y* axis) and a 1.75-fold increase in apparent bead size along the *Z* axis size was observed for beads below microarray substrate and graphene electrode.

We investigated light-induced artifacts in graphene and gold electrodes in vitro using a standard OGs setup (fiber-coupled light emitting diode (LED) at 470 nm) (Supplementary Fig. 6). Gold electrodes exhibit prominent light-induced artifacts during OG stimulation. Supplementary Fig. 7A shows typical light-induced artifacts recorded by gold electrodes for light intensities from 6.4 to 54.1 mW mm^−2^ with a fixed illumination time of 20 ms. When the same experiment was repeated with transparent graphene electrodes, no measurable artifacts were detected (Supplementary Fig. 7B). To further inspect the artifacts of gold and graphene electrodes in the frequency domain, repeated 20 ms light pulses were applied to the electrode site at 10 Hz and the power spectrum was plotted for both recordings (Supplementary Fig. 7C, D). For the gold electrode, we observed a 10 Hz peak corresponding to the artifact signals induced by light stimulation at this frequency. Besides the 10 Hz artifacts, some higher-order harmonic signals at 20, 30, and 40 Hz also existed in the recordings. For the graphene electrode, there were no detectable artifact components within the 0–60 Hz range. We performed an additional in vitro test with graphene microarrays using a 473 nm laser, which illuminated the array through a 20× microscope objective (same setup as for in vivo experiments described in Methods). The laser beam had a diameter of 230 µm (FWHM) and fully covers the area of one graphene electrode. For all tested light intensities up to 240.7 mW mm^−2^, illumination of the substrate next to a graphene electrode does not elicit any detectable artifacts. When the laser illuminated a graphene electrode directly, artifacts were observed at light intensities higher than 60 mW mm^−2^ (Supplementary Fig. 7E). These experiments suggest that transparent graphene electrodes can be safely used for in vivo OG stimulation and electrical recording experiments, ensuring crosstalk-free operation. After confirming artifact-free operation under in vitro conditions, we explored the capabilities of simultaneous optical imaging, OG stimulation, and electrical recordings through transparent graphene electrodes in vivo in the primary somatosensory cortex of anesthetized mice.

### Deep in vivo 2-photon imaging through graphene arrays

**Fig. 2:**
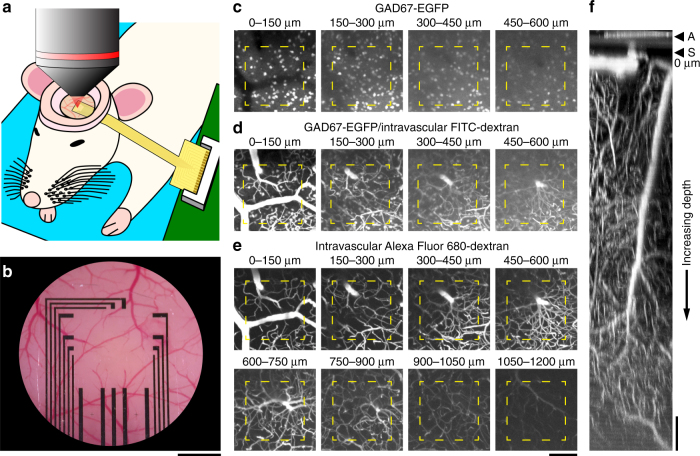
Structural 2-photon imaging through a graphene microelectrode array. **a** After placement of head-post (not shown), craniotomy, and dura removal, the graphene microelectrode array is placed on the surface of the primary somatosensory cortex (SI). The exposure is covered with agarose and closed with a coverslip. Dental acrylic is used to fix the coverslip and the arrays’ connecting wires to reduce motion. **b** Bright-field image of an exposure with graphene microelectrode array. Graphene electrodes and wires in the center of the array are not visible, but the gold wires for connection to the amplifier board. Scale bar, 500 µm. **c** Detection of EGFP-expressing interneurons in GAD67-GFP mice below a single graphene electrode (yellow outline). Images were acquired using 2-photon excitation at 905 nm (laser power from 3 mW at the surface to 57 mW at 600 µm cortical depth). **d** The same region as in **c** is shown, but after intravascular injection of FITC-dextran (2 MDa); images were acquired using 2-photon excitation at 950 nm (laser power from 8 mW at the surface to 77 mW at 600 µm cortical depth). **e** The same region as in **c**, **d** is shown, but after additional intravascular injection of Alexa Fluor 680-dextran (2 MDa); images were acquired using 2-photon excitation at 1280 nm (laser power from 2 mW at the surface to 44 mW at 1200 µm cortical depth). **c**–**e** Show maximum intensity projections (MIPs) of images acquired at different depths (step size: 3 µm) below the cortical surface (MIP range is indicated above individual images). Scale bar for **c**–**e**, 100  µm. **f** Orthogonal (XZ) MIP of the Alexa Fluor 680-dextran dataset shown in **e**; graphene microelectrode array (A) and cortical surface (S) are on the top of the image. Scale bar, 100 µm

To demonstrate deep 2-photon imaging through graphene electrodes in vivo, we placed the transparent array onto the exposed cortical surface, placed a glass coverslip on top, and sealed the window (Fig. 2a, b; see Methods). We used 2-photon imaging in frame-scan mode to acquire image stacks, i.e., a series of images in the horizontal (*XY*) plane parallel to the cortical surface, with individual images spacing 3 μm along the depth (*Z*) axis. Figure 2c–f illustrates data from image stacks from the cortical surface down to 1200 μm in a GAD67-GFP transgenic mouse expressing enhanced green fluorescent protein (EGFP) in all inhibitory (GABAergic) cortical neurons^10^. EGFP was excited at its peak resonance of 905 nm and detected with a 490–560 nm bandpass filter (Fig. 2c). Next, we injected a bolus of 2-MDa fluorescein-isothiocyanate (FITC)-dextran as intravascular tracer (see Methods) and acquired an image stack, while exciting at 950 nm (Fig. 2d). This illumination wavelength excited both FITC and EGFP providing a compromise between efficiency of excitation (peak excitation of fluorescein is at 800 nm) and penetration of the excitation beam in tissue (950 nm penetrates better than 800 nm). FITC and EGFP have overlapping emission spectra and were detected in the same photomultiplier (PMT) channel.

To explore the possibility of deeper imaging through the graphene array, we injected an additional bolus of the intravascular tracer Alexa Fluor 680-dextran and switched the excitation to 1280 nm, which is the peak resonance of Alexa Fluor 680^11^. This illumination wavelength lies within one of the spectral windows of opportunity, where combined effects of scattering and absorption are relatively low, and therefore allowing penetration of light through tissue. Alexa Fluor 680 was detected using a 954 nm shortpass filter. Under this regime, we could image down to 1200 μm below the cortical surface covered with the graphene array. Figure 2e–f show that our graphene electrodes provide virtually no obstacle for deep 2-photon imaging. That methodology can directly be applied to various research problems involving monitoring cortex-wide activation, while probing the activity at deeper layers.

### Simultaneous electrical recording and 2P Ca^2+^ imaging

**Fig. 3:**
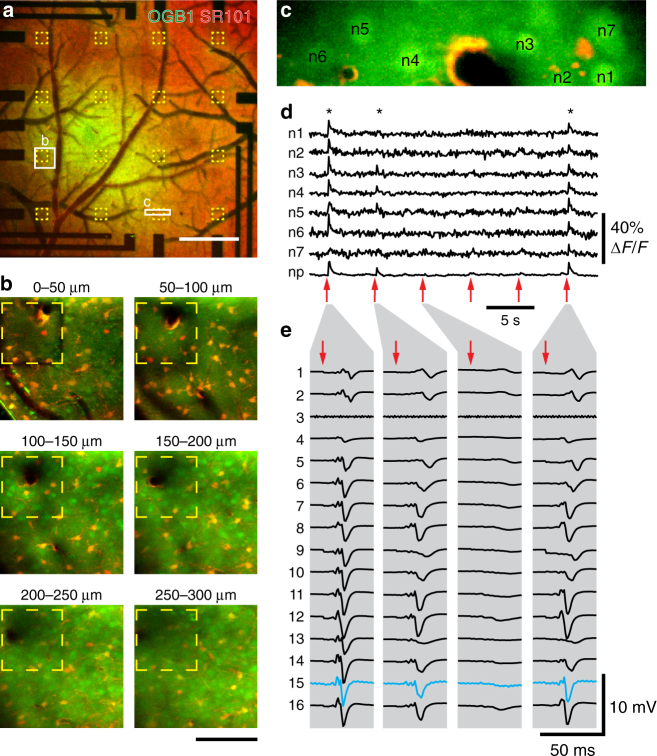
Combination of 2-photon-based calcium imaging with electrical recordings. Before placement of the graphene microelectrode array, the calcium indicator Oregon Green 488 BAPTA-1 (OGB1) AM ester was pressure-microinjected and astrocytes were stained with Sulforhodamine 101 (SR101). **a** Overview image of OGB1 (green) and SR101 (red) staining below the graphene microelectrode array. Yellow outlines indicate single graphene electrodes, white rectangles indicate imaging sites shown in **b** and **c**. Scale bar, 500 µm. **b** Two-photon imaging of OGB1 (green) and SR101 (red) below a single graphene electrode (yellow outline). Maximum intensity projections (MIPs) of images acquired at different depths (step size: 3 µm) below the cortical surface are shown (MIP range indicated above individual images). Scale bar, 100 µm. **c** Image of the region (size: 140 µm × 32.6 µm) used for functional Ca^2+^ imaging as shown in **d**. In the center, a vessel (dark) passes the imaging plane. For analysis, images were segmented into individual neurons (n1–n7; bright OGB1, no SR101 staining), astrocytes (bright OGB1 and SR101 staining), and neuropil (OGB1 staining, no SR101 staining). **d** Calcium traces (OGB1 fluorescence change relative to pre-stimulus baseline, Δ*F*/*F*) of individual neurons (n1–n7 as shown in **c**) and neuropil (np). Single electric stimuli (300 µs, 1 mA) were delivered every 5 s (red arrows) to the whisker pad. Imaging was performed 150 µm below the cortical surface at 12 Hz with 2-photon excitation at 800 nm and a power of 50 mW. Asterisks (*) indicate “responsive” trials. **e** Corresponding electrical responses to electrical whisker pad stimulation (red arrow) measured with graphene microelectrode array; traces from four individual trials matching stimulations no. 1, 2, 3, and 6 in **d** are shown. Graphene electrodes are numbered 1 (top left in **a**) to 16 (lower right in **a**); electrode 3 is non-functional and 2-photon Ca^2+^ imaging was performed right below electrode 15 (highlighted in blue), leading to slightly increased noise in this channel. Artifacts resulting from electrical stimulation were removed by linear interpolation

**Fig. 4:**
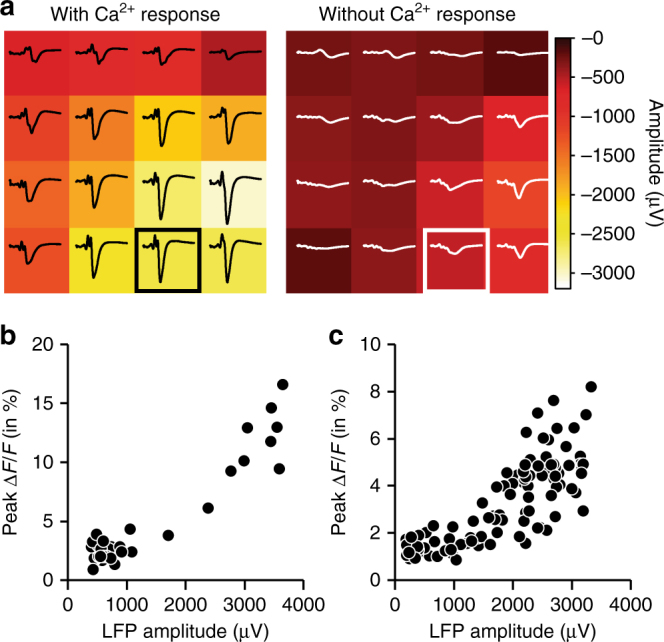
Relationship between local field potential (LFP) and Ca^2+^ transients. **a** Comparison of LFP amplitudes across the array in trials where Ca^2+^ transients were observed (left; 10 trials) or not observed (right; 13 trials, only trials with LFP response amplitudes < − 500 µV are included); amplitudes (heatmap) and traces are trial averages; channel 15 (where Ca^2+^ data was acquired) is highlighted. Data from experiment shown in Fig. 3c–e. **b**, **c** Correlation between LFP and Ca^2+^ peak amplitude per trial; trials with LFP responses < − 400 µV are included. Calcium signal amplitudes are derived from two different animals; representative experimental data for **b** (37 trials included) is shown in Fig. 3, representative data for **c** (100 trials included) is shown in Supplementary Fig. 8

We combined LFP measurements using the transparent graphene array with 2-photon calcium imaging of neuronal activity. We injected the calcium indicator Oregon Green BAPTA-1 (OGB1) into cortical layer II/III of the primary somatosensory cortex and applied Sulforhodamine 101 (SR101) to label astrocytes^12–14^ before placing the graphene microelectrode array on the cortical surface and sealing the window (Fig. 3a, b). To stimulate neuronal activity, we used a single electrical pulse (300 μs, 1 mA) delivered to the contralateral whisker pad. Fields-of-view (FOVs) of ~ 50 μm × 100 μm were imaged at ~ 10 Hz in frame-scan mode. Each FOV, including multiple neuronal cell bodies, was imaged continuously for ~ 250 s. During this time, we delivered 50 stimuli at an interval of 5 s. An example FOV is shown in Fig. 3c; neuronal cell bodies (labeled n1–n7) are brighter than the surrounding neuropil; the FOV also contains a vessel (black) with adjacent astrocyte labeled both with OGB1 and SR101 (yellow). Stimulus-induced calcium increases were observed in neuronal cell bodies and neuropil for some of the stimulus trials (labeled by asterisks in Fig. 3d; recordings of all six trials in Supplementary Movie 1). For the same “responsive” trials, a robust LFP response was detected by all working electrodes in the array (Fig. 3e). In a more detailed analysis, we found that LFP responses have large amplitudes and are spatially broad in trials where Ca^2+^ responses are observed, whereas in trials without detectable Ca^2+^ response, LFP responses are comparatively weak and more localized (Fig. 4a and Supplementary Movie 1). Furthermore, we found a positive correlation between LFP amplitude (from channels above the FOV or next to the FOV where calcium data were recorded) and Ca^2+^ signal amplitude (Fig. 4b, c), which is consistent with previous reports in the literature^15^. Results obtained in another preparation are shown in Supplementary Fig. 8. These experiments and additional data shown in Supplementary Fig. 9 demonstrate that transparent graphene electrodes are well suited for simultaneous in vivo 2-photon imaging providing sufficient transparency at the operating spectra and offering simultaneous 2-photon imaging and electrical recordings with no photovoltaic artifacts. Importantly, successful LFP recordings were obtained even with the graphene electrode directly above the imaging FOV (blue trace in Fig. 3e); virtually artifact-free recording was achieved, as evident from the comparison of SNR for blue and black traces. This combined methodology can be used to investigate correlations between field potentials or cortical rhythms and dynamic cellular calcium responses.

### Simultaneous electrical recordings, 2P imaging and optogenetics

**Fig. 5:**
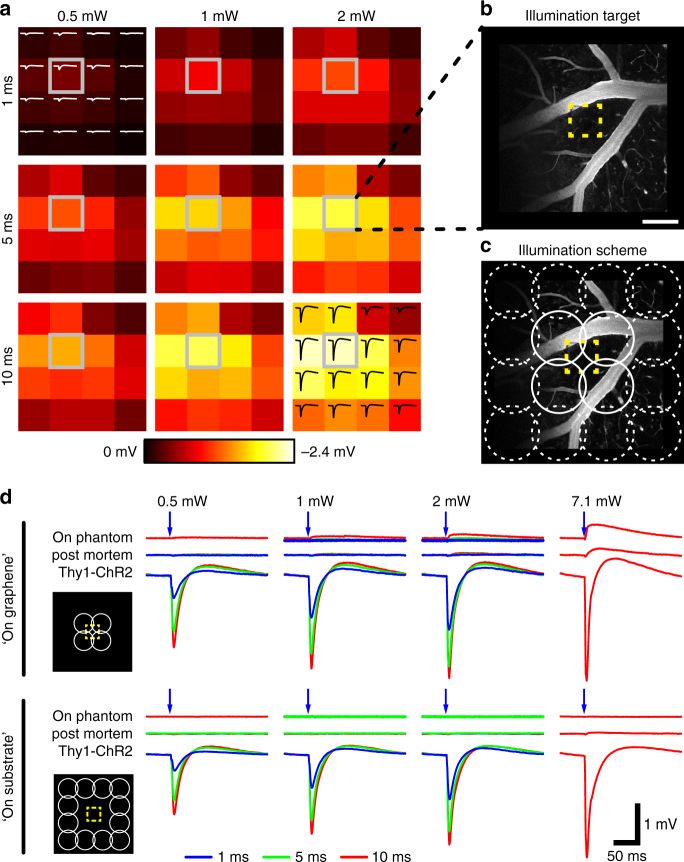
Measurement of optogenetic photostimulation-induced electrical potentials in Thy1-ChR2 mice that express channelrhodopsin-2 (ChR2) in layer-V pyramidal neurons. **a** A 473 nm laser beam (diameter: 210 µm full-width half-maximum) was sent through a 20× objective next to one graphene electrode (highlighted in gray). Different illumination times (1, 5, and 10 ms) and powers (0.5, 1, and 2 mW) were used. Heatmaps represent maximal response amplitude of the 16 electrodes in the array; example traces are shown for 0.5 mW/1 ms (top left) and 2 mW/10 ms (bottom right). **b** Two-photon image of the illumination target after intravascular injection of FITC-dextran (2 MDa); the yellow outline indicates the position of the graphene electrode. Scale bar, 100 µm. **c** Per condition, illumination with the 473 nm laser beam was targeted to 12 sites “on substrate” (circles with broken lines) and to four sites “on graphene” (circles with closed lines) where the laser beam directly irradiates part of the electrode (yellow outline). **d** Electrical response of the single graphene electrode within the illumination target in a live Thy1-ChR2 mouse (“Thy1-ChR2”), in the same animal after death (“post mortem”), and in vitro on an agarose phantom (“on phantom”). Laser pulses of 1, 5, and 10 ms length with powers of (0.5, 1, 2, and 7 mW) were used. Average responses of 12 “on substrate” or 4 “on graphene” illuminations per condition are shown; averaged responses from “on substrate” illuminations were used for response amplitude comparison across the microelectrode array as shown in **a**

Crosstalk-free integration of optical imaging, OGs, and electrophysiological recordings is critical for investigating neural activity on a circuit or population level, whereas, at the same time, examining the causal role of individual neurons or groups of neurons in circuit function. We investigated the performance of graphene electrode arrays in studies involving both 2-photon imaging and single-photon OG photostimulation. To this end, we performed electrical recordings with graphene arrays and OG stimulation via a cylinder-shaped blue (473 nm) laser beam delivered through the microscope objective with ~ 210 μm in diameter (FWHM); this stimulation protocol has been used in a recent study from one of our laboratories^16^. Experiments were performed in Thy1-ChR2 transgenic mice expressing the OG actuator channelrhodopsin-2 (ChR2) in layer V pyramidal neurons (Fig. 5). We illuminated the cortical surface at one graphene electrode with three different laser powers (0.5, 1, and 2 mW) for 1, 5, and 10 ms. The LFP responses to OG stimulation scale with power and duration of the laser pulse (Fig. 5a); the strongest response was recorded at the “targeted” electrode and amplitudes decline with distance from the illumination target. To investigate artifacts resulting from direct illumination of the graphene electrode with the 473 nm laser, we tested two illumination geometries: overlapping with the recording graphene electrode (“on graphene”) vs. in-between electrodes (“on substrate”) (Fig. 5b, c). We used four laser powers (0.5, 1, 2, and 7 mW) and illumination times of 1, 5, and 10 ms and performed the same OG stimulation protocol in vivo, post mortem, as well as with arrays placed on agar phantoms (blocks of 2% agar in artificial cerebrospinal fluid (ACSF)). Virtually, no artifact was observed at laser powers ≤ 2 mW or 50 mW  mm^−2^ (Fig. 5d; see Supplementary Fig. 7E for in vitro results), whereas light-induced potentials were clearly visible upon illumination of the electrode but not the surrounding substrate with a laser power of 7.1 mW (or 210 mW mm^−2^), which is considerably higher than the power typically used for OG photostimulation^16,17^.

**Fig. 6:**
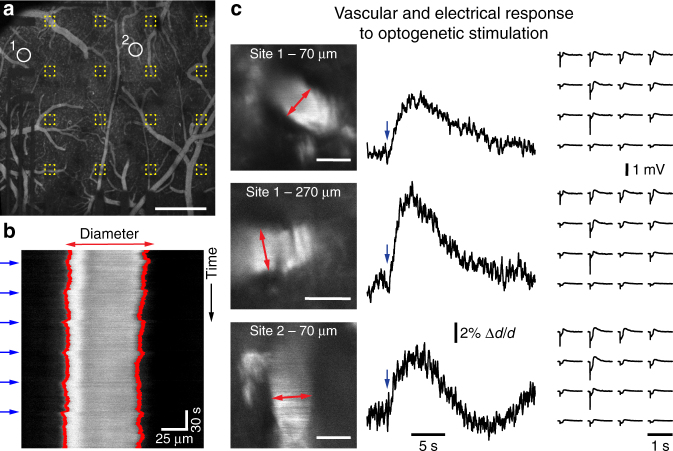
Measurement of vascular responses to optogenetic (OG) photostimulation below graphene microelectrode arrays in Thy1-ChR2 mice. **a** Location of diving arterioles for diameter measurements with 2-photon imaging as shown in **b**, **c**. Yellow outlines indicate single graphene electrodes. Data was acquired after intravascular injection of FITC-dextran (2 MDa). Scale bar, 500 µm. **b** Line-scan mode was used to measure time courses of single arteriole diameters; blue arrows indicate when a 473 nm laser stimulus was delivered, red lines indicate computed vessel borders used for estimation of diameter changes. **c** High-magnification scans of arteriole segments are shown on the left (scale bars: 50 µm); red arrows indicate line scan location. Vascular responses (diameter change relative to pre-stimulus baseline, Δ*d*/*d*) averaged from five to six photostimulation events (indicated by blue arrow) are shown in the middle; respective electrical responses to photostimulation measured with the graphene array are shown on the right. Note that stimulus intensities and illumination times used to elicit detectable vascular responses (50–100 ms at 7 mW) are larger than photostimulation shown in Fig. 5. Control experiment with a ChR2-negative animal is shown in Supplementary Fig. 10

Next, we performed simultaneous LFP measurements and 2-photon imaging of arteriolar dilation induced by OG photostimulation in Thy1-ChR2 mice. Therefore, 2 MDa FITC-dextran was injected as intravascular tracer (Fig. 6a) and line-scan imaging was used to follow changes in arteriolar diameter. Here, transitions between (dark) background and (bright, FITC-dextran-filled) intravascular lumen are used to estimate vessel diameters and their dynamic changes in response to OG stimulation (Fig. 6b, c). Consistent with our previous reports, OG stimulation resulted in robust arteriolar dilation, mediated through messengers released by neurons in response to OG stimulation that are sensed by cells of the vasculature^16^. To exclude that vascular responses were elicited through tissue heating (see ref. ^18^), especially due to light absorption by the microarray, we applied the same OG stimulus in a ChR2-negative mouse and did not observe any stimulus-induced changes of arteriole diameters, which were measured close to the cortical surface (Supplementary Fig. 10). Simultaneously obtained surface LFP recordings exhibited clear responses to the OG stimulus (Fig. 6c). To elicit detectable vascular responses, intensity and duration of the OG stimulus (illumination for 50–100 ms at 7 mW) were relatively high (and larger compared to stimuli in Fig. 5a), so that the spatial distribution of measured LFP responses became almost independent from the location of OG photostimulation (compare spatial LFP response profiles upon OG stimulation at site 1 and site 2 in Fig. 6c). These data demonstrate for the first time (to the best of our knowledge) that transparent graphene electrode array technology can be successfully employed in combination with both 2-photon imaging and single-photon OG photostimulation without causing any crosstalk between three modalities.

### Simultaneous electrical recordings and hemodynamic imaging

**Fig. 7:**
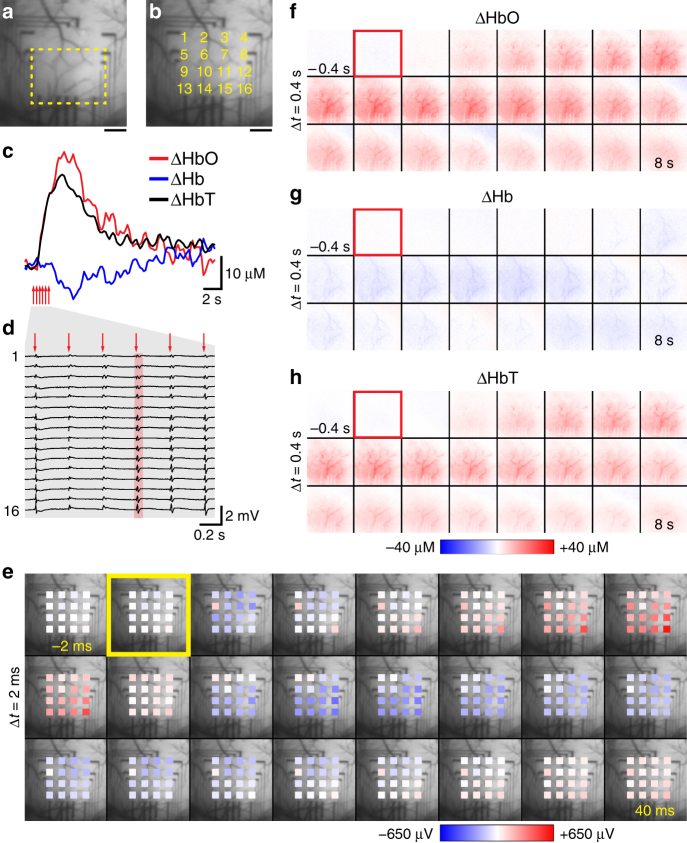
Combination of macroscopic hemodynamic optical imaging with electrical recordings. Light absorption at six wavelength ranges (560–610 nm, 10 nm step size) was recorded with a CCD camera at an acquisition frequency of ca. 18 Hz. Absorption at these wavelengths was converted to concentrations of oxyhemoglobin (HbO), deoxyhemoglobin (Hb), and total hemoglobin (HbT) measured as changes relative to pre-stimulus baseline. A train of six electric pulses (1 mV for 2 s at 3 Hz, pulse width: 300 µs) was delivered to the whisker pad. **a** Image of the exposure and graphene microelectrode array with region of interest (yellow line) used for evaluation of hemodynamic signals. **b** Image of the exposure with location and numbering of graphene electrodes. Scale bars for **a** and **b**, 500 µm. **c** Changes in HbO, Hb, and HbT in response to electrical whisker pad stimulation (average of 10 trials across the region of interest shown in **a**). **d** Corresponding recordings from the graphene microelectrode array; voltage traces recorded during stimulation period are shown (average of 10 trials; channel positions as indicated in **b**). **e** Spatiotemporal analysis of the electrical response to one electrical stimulus (red highlight in **d**). The sequence of 24 images covers a recording period of 42 ms (time between two images: 2 ms); the yellow rectangle indicates stimulus delivery. **f**–**h** Spatiotemporal analysis of changes in HbO, Hb, HbT in response to whisker pad stimulation. The sequence of 24 images covers a recording period of 8.4 s (time between two images: 400 ms); red rectangles indicate stimulation onset

Comprehensive investigation of brain activity often requires bridging between measurements on different scales. Surface LFP recordings can provide such a bridge when combined with both micro- and mesoscopic optical measurements. To this end, we sought to demonstrate the utility of graphene devices for integration with camera-based mesoscopic imaging of intrinsic hemodynamic signals. We used spectral imaging of oxyhemoglobin (HbO), deoxyhemoglobin (Hb), and total hemoglobin (HbT) to detect stimulus-induced cortical hemodynamic activity (as a combination of changes in cerebral perfusion and oxygen metabolism), while imaging through a graphene surface array for simultaneous LFP recordings (Fig. 7). For spectral imaging of hemoglobin oxygenation and its change in response to neuronal activation, light from a tungsten-halogen light source was filtered through a rotating filter wheel with individual filters ranging from 560 to 610 nm; illumination of the graphene array had no influence on LFP recordings (not shown). Six electrical 300 μs pulses delivered to the contralateral whisker pad over 2 s (at 3 Hz) induced neuronal activity and a hemodynamic response. The hemodynamic response measured below the graphene array (see overview in Fig. 7a, b) is characterized by a rapid HbO increase 1 s after stimulus onset and a delayed decrease in Hb and takes about 20 s to return to baseline (Fig. 7c); Fig. 7f–h show the spatiotemporal dynamics of HbO, Hb, HbT changes (see also Supplementary Movie 2). LFP responses to every of the six electrical stimuli are separated in time and show different amplitudes in different channels, i.e., across space (Fig. 7d; electrical recordings also in Supplementary Movie 3). The center of neuronal activity with largest LFP amplitudes (lower right corner of the array in Fig. 7e) overlaps spatially with the initial (< 2 s after stimulus onset) hemodynamic response (Fig. 7f–h). Thus, this data shows that transparent graphene technology provides a versatile platform for combination with a suite of optical tools for imaging and manipulation of cortical activity across scales, ranging from monitoring of cellular activity with 2-photon microscopy to large-scale optical monitoring of hemodynamic response.

## Discussion

Transparent graphene array technology provides a viable complementary alternative to needle arrays for multimodal measurements/manipulations within the penetration depth of multiphoton microscopy. In cases where depth-resolved electrical recordings are not required, optically transparent graphene surface arrays allow seamless integration with depth-resolved optical imaging modalities while circumventing the need to insert invasive probes into brain tissue. This technology is also well-suited for neurovascular and neurometabolic studies providing a “gold standard” neuronal correlate for optical measurements of vascular, hemodynamic, and metabolic activity.

Advancements in measurement technology play a critical role in neuroscience enabling scientific inquiry and powering discovery. This is also the goal of the ongoing BRAIN Initiative^19,20^. Several recent publications have demonstrated successful combination of electrode array recordings with OG photostimulation through integration of optical waveguides or incorporation of LEDs along penetrating electrode shanks^21–26^. As a further step in this direction, ongoing efforts are focused on the addition of photodetectors along the electrode shanks that would provide optical imaging capability alongside with electrical recordings and OG photoactivation^21^. This strategy is particularly attractive for deep brain studies targeting beyond the penetration limits of multiphoton microscopy^27,28^. Within these limits, however, simultaneous electrical recordings and optical imaging/manipulation can be achieved by engineering optically transparent electrode arrays^2,3,29–31^. To this end, in the present study, we introduced a new transparent graphene microelectrode array and demonstrated its unique capabilities for integration with 2-photon imaging and single-photon OG photostimulation.

In this study, transparent graphene array technology has been significantly advanced beyond previously reported transparent electrode demonstrations by incorporating a new graphene-friendly fabrication process. This new fabrication process avoids any crack formation in the transfer process proving 95–100% yield for the electrode arrays. Such techniques can as well be directly employed to fabricate high-density, large-area transparent arrays to monitor brain-scale cortical activity in large animal models. The fabrication process is also complementary metal-oxide-semiconductor (CMOS)-compatible, so that graphene arrays can be directly integrated with amplifying and multiplexing circuits on the same chip^32^.

With this technology, we demonstrate simultaneous mapping of surface LFP and high-resolution 2-photon imaging of neuronal calcium transients. LFP signals recorded from the cortical surface reflect flow of currents along the vertically aligned pyramidal cells’ apical dendrites, largely produced by synaptic inputs. Therefore, these signals provide a measure of circuit activity^33,34^. Thus, combination of surface LFP recordings with 2-photon imaging of neuronal calcium would allow investigation of spiking activity in specific neurons (resolved with 2-photon imaging) in the context of circuit behavior, e.g., “up” and “down” states^35,36^.

This combination of measurements is also of relevance for studies aiming to bridge neuronal activity across scales and ultimately connect the large body of classical neuroscience knowledge, obtained from research in model systems (cell cultures, brain slices, in vivo mouse recordings, etc.), to human noninvasive electro-/magnetoencephalography measurements^1^. Likewise, simultaneously acquired surface LFP and 2-photon measurements of single-vessel dilation as well as mesoscopic measurements of cortical hemodynamics would help neurovascular studies aiming towards the physiological underpinning of human noninvasive functional magnetic resonance imaging (fMRI) signals^37^.

With rapid advancement in multiphoton microscopy and a growing arsenal of synthetic and genetically encoded optical probes, we envision that recordings with optically transparent graphene electrode arrays will be combined with a wide range of microscopic physiological parameters related to neuronal activity^38,39^, glial function^40^, vascular dynamics^16,37^, immune response^41^, energy consumption^42^, and more. With the current trends to move away from anesthesia and towards longitudinal imaging in awake behaving mice^43^, incorporation of graphene surface arrays within a chronic cranial window implant^44^ would facilitate the adoption of our technology. To this end, ongoing efforts in our laboratories are focused on engineering such double-duty implants providing a transparent optical window and capability of space-resolved surface LFP measurements. For multiphoton imaging, further improvement of image quality can be achieved by addition of adaptive optics to correct for distortions of the excitation wavefront due to mismatched refractive indices between graphene/PET substrate and cortical tissue^45^.

Compatibility with large FOV imaging, exemplified in previous studies^2,3,31^ by combination with charge-coupled device (CCD)-based imaging of vascular/hemodynamic signals, can provide an intermediate step in bridging brain phenomena across scales. Scaled up to cover a larger cortical area, our technology may also be very informative for studies of large-scale, multi-area neuronal activity/connectivity^46^, as well as for neuronal underpinning of large-scale spontaneous hemodynamic oscillations and their correlation across different regions of the cerebral cortex^47^. As a nonmagnetic material, graphene array technology can also be utilized to develop fMRI-compatible implantable arrays.

To conclude, we envision that our transparent graphene-based electrode array technology will find application in multiple areas, advancing our understanding of how microscopic neuronal activity at the cellular scale translates into macroscopic activity of large neuronal populations and providing a neuronal correlate for optical measurement of vascular, hemodynamic, or metabolic activity.

## Methods

### Graphene transfer

The bubbling transfer method^9^ was adopted to protect graphene from cracking during the process. Therefore, 300 nm-thick 495 PMMA A4 was spin-coated on the graphene/copper bilayer structure. This PMMA/graphene/copper tri-layer was then connected to the cathode of a 20 V DC power supply, whereas the anode was submerged in 0.05 M NaOH in deionized water. As the trilayer was gently and gradually immersed into the NaOH solution, hydrogen gas bubbles formed in between the graphene and copper layer, and exfoliated PMMA/graphene from the copper foil. The graphene layer needed to be thoroughly cleansed by floating on the surface of deionized water for three times before it was placed onto the gold pads on the flexible substrate.

### Four-step cleaning procedure for photoresist removal

Graphene electrode impedance and susceptibility toward light-induced artifacts are highly dependent on photoresist residue. Therefore, we developed a four-step stripping/cleaning protocol to remove organic residues from the graphene surface that was crucial to achieve arrays with low impedance at high yield. After oxygen plasma etching, the sample was soaked in AZ 1-Methyl-2-pyrrolidon and Remover PG to remove AZ1512 and PMGI, respectively. Another acetone bath was used to remove Remover PG residue. All three rinses were conducted at room temperature with gentle agitation. The last cleaning step included ten cycles of isopropanol/deionized water rinse.

### Graphene microelectrode array fabrication

Using the new graphene transfer and photoresist removal methods, we developed the following fabrication process (see also Fig. 1b). Fifty-micrometer-thick clear PET (Mylar 48-02F-OC, elastic modulus: 4.9–5.1 GPa) is used as substrate for its high optical transmittance. To provide mechanical support during fabrication, 20 µm-thick PDMS was spin-coated on a 4-inch silicon wafer, and PET film was placed on the PDMS adhesive layer. Ten nanometer of chromium and 100 nm gold were deposited onto the PET film with a Denton Discovery 18 Sputter System. Metal wires were patterned with photolithography and wet-etching. Graphene was then transferred onto the designed area as described above. The device was dried completely at room temperature first and baked at 125 ˚C for 5 min to enhance bonding between graphene and PET substrate. The PMMA scaffold was removed in a room-temperature acetone bath for 20 min, following by 10 cycles of isopropanol/deionized water rinse. Graphene contact pads were patterned with PMGI/AZ1512 bilayer photolithography and oxygen plasma etching (Plasma Etch PE100), followed by the four-step cleaning method as described above. Finally, an 8 µm-thick SU-8 2005 encapsulation layer with openings only at active electrode areas was patterned with photolithography, followed by 10 cycles of isopropanol/deionized water rinse to cleanse SU-8 residue.

### Animal procedures

All experimental procedures were performed in accordance with the guidelines established by the UCSD Institutional Animal Care and Use Committee. We used 9 adult mice of either sex including three Thy1-ChR2-YFP (Jackson Stock Number 007612; heterozygous on a mixed C57Bl6/ICR background), two GAD67-GFP^10^, and six wild-type ICR mice. Surgical procedures in mice expressing ChR2 were performed in a dark room using a 515 nm long-pass filter (Semrock) in the surgical microscope light source to avoid OG photostimulation during installation of the cortical window. Mice were anesthetized with isoflurane during surgical procedures (5% initially, 1–1.5% during all procedures). A catheter was inserted into the femoral artery. A metal holding bar was glued to the temporal bone for immobilization of the head during imaging. A ~ 4 × 4 mm cranial window was centered on the whisker area of primary somatosensory cortex; overlying skull contralateral to the holding bar was exposed and dura mater removed.

In calcium imaging experiments, 50 μg calcium indicator Oregon Green 488 BAPTA-1 AM ester (OGB1; O-6807, Thermo Fisher) was first dissolved in 4 μl of 20% Pluronic F-127 in dimethyl sulfoxide (P3000MP, Thermo Fisher); 80 μl of ACSF were added to the OGB1 solution to yield a final concentration of 0.5 mM OGB1. OGB1 solution was pressure-microinjected into the cortical tissue^13^ within the whisker area of the somatosensory cortex. SR101 (S7635, Sigma) in ACSF was applied topically for ~ 2 min to label astrocytes^12^, providing a contrast in tissue that was used for visual assessment of potential damage due to experimental procedures. Excess dye was washed away with ACSF.

The graphene array was placed on the cortical surface, a drop of 0.7% (w/v) agarose (A9793, Sigma) in ACSF was applied on top of the array, and the exposure was covered with a rectangular glass coverslip and sealed with dental acrylic. To avoid herniation of the exposed brain due to excessive intracranial pressure, the dura mater over the IVth cerebral ventricle was punctured, thus allowing drainage of CSF. After the exposure was closed, the drainage hole was sealed with agarose.

After closing the exposure, mice were left to rest under 1% isoflurane for 45 min, which minimized leakage of drugs onto the exposed cortical tissue through cut dural blood vessels. Then, isoflurane was discontinued and anesthesia was maintained with α-chloralose (50 mg kg^–1^ h^–^^1^, C0128, Sigma or 100459, MP Biochemicals). Mice were paralyzed with pancuronium bromide (0.4 mg kg^–^^1^ h^–^^1^, P1918, Sigma)^48^, and ventilated (~ 110 min^−1^) with 100% O_2_. α-chloralose, pancuronium bromide, or 5% dextrose in saline were supplied through the femoral line every 30 min for the duration of data acquisition. Expired CO_2_ was measured continuously using a micro-capnometer (Cl240, Columbus Instruments). Heart rate, blood pressure, and body temperature were monitored continuously. Blood gas was analyzed to cross-validate micro-capnometer measurements. Respiration was adjusted to achieve a PaCO_2_ between 30 and 40 mm Hg and pH between 7.35 and 7.45.

For vascular imaging experiments, 2 MDa dextran-conjugated FITC (FD-2000S, Sigma) or 2 MDa dextran-conjugated Alexa Fluor 680 were injected through the femoral line (50–100 μl of 5% (w/v) solution in PBS). To prepare 2 MDa Alexa Fluor 680-dextran, Alexa Fluor 680 NHS ester (A20008, Thermo-Fisher) was conjugated to 2 MDa amino dextran (AD2000x150, Finabio) using a custom conjugation protocol that can be found on the Devor lab academic website http://nil.ucsd.edu/ under “Resources”.

### Two-photon imaging

Images were obtained using an Ultima 2-photon laser scanning microscopy system from Bruker Fluorescence Microscopy (formerly Prairie Technologies) equipped with an Ultra II femtosecond Ti:Sapphire laser (Coherent) tuned to 905  nm for imaging of EGFP and 950 nm for imaging of FITC. For penetration deeper than ~ 600 μm, an optical parametric oscillator (Chameleon Compact OPO, Coherent) pumped by the same Ti:Sapphire laser was tuned to 1280 nm. The OPO was used in conjunction with intravascular administration Alexa Fluor 680-dextran^11^. FITC, EGFP, and Alexa Fluor 680 were imaged using cooled GaAsP PMT tube detectors (H7422P-40, Hamamatsu). SR101 was imaged using a multialkali PMT (R3896, Hamamatsu).

In experiments involving OG stimulation, the main dichroic mirror contained a 460–480 nm notch (Chroma ZT470/561/NIR TPC). An additional filter that blocks wavelengths in between 458 and 473 nm (Chroma ZET458-473/561/568/NIR M) was added in front of the PMT block. Nevertheless, residual bleed-through of the 473 nm light prevented us from using GaAsP PMT detectors. Therefore, in these experiments, FITC was imaged using a multialkali PMT. For imaging Alexa Fluor 680-dextran, custom-made filters (Chroma) were used; the main dichroic mirror contained a 440–480 nm notch and allowed light transmission from 780 to 1400 nm, the corresponding filter in front of the PMT blocks light between 440 and 480 nm, and above 750 nm.

We used 4× (Olympus XLFluor4x/340, numerical aperture (NA) = 0.28) or 5× (Zeiss Plan-NEOFLUAR, NA = 0.16) objectives to obtain low-resolution images of the exposure. Olympus 20× (XLUMPlanFLNXW, NA = 1.0 and UMPlanFI, NA = 0.5) water-immersion objectives were used for high-resolution imaging. The laser beam diameter was adjusted to overfill the back aperture. Arteriolar diameter measurements were performed in a “free-hand” line-scan mode with a scan rate of 25–50 Hz. The scan resolution was 0.5 μm or less. Calcium imaging was performed in frame-scan mode at ~ 10 Hz per frame.

### Sensory stimulation

Electrical stimulation was delivered to the whisker pad contralateral to the cortical exposure through a pair of thin needles inserted under the skin using 300 µs, 1 mA electrical pulses. For calcium imaging, 50 trials with 5 s interstimulus interval (ISI) were delivered at each measurement location. For mesoscopic imaging of cortical hemodynamics, a train of six electrical pulses (300 µs, 1 mA) were delivered at a rate of 3 Hz with an ISI of 30 s.

The stimulation device (A365 stimulus isolator, WPI) was triggered using a separate PC that also acquired timing signals for data acquisition (“trigger out” signals for each frame/line) and physiological readings using a National Instruments IO DAQ interface (PCI-6229) controlled by custom-written software in MATLAB (MathWorks, Inc.). The timing of electrophysiological acquisition as well as each optical frame/line relative to the stimulus onset was determined during data analysis based on acquired triggering signals.

### OG stimulation

In 2-photon experiments, OG stimulation was delivered though the objective using a 473 nm cylinder-shaped CW laser beam ~ 210 μm in diameter (FWHM)^16^. The beam was directed to defined locations in the sample using a dedicated set of galvanometer mirrors. The duration of the light pulse was controlled by a dedicated shutter and synchronized with imaging. Laser power was measured by directing the entire beam into the sensor of a broadband power meter (13PEM-001, Melles-Griot). The beam diameter was measured with a CCD-based laser beam profiler (LBP-2, Newport).

### Recording and analysis of electrophysiological data

The electrophysiological data were recorded by the RHD2000 amplifier board and the RHD2000 evaluation system from Intan Technologies. The sampling rate was 10 kHz. The DC offset was removed by the built-in filters of the system. Data were analyzed in MATLAB using custom-written software. After import and conversion into MATLAB files, trigger signals were detected, artifacts from electrical stimulation were removed by linear interpolation, if necessary, and the sampling rate was reduced to 1 kHz for trial average and further processing. For normalization, data recorded 50 ms before stimulus delivery were averaged and used as baseline value.

### Analysis of imaging data

Data were analyzed in MATLAB using custom-written software as described in^16,37,40^. For analysis of calcium imaging data, neuronal cell bodies were segmented from composite red (SR101) and green (OGB1) images. For individual regions of interest (ROIs), the calcium signal per frame was calculated as average OGB1 fluorescence of all pixels within the ROI. This calculation was repeated for each frame in the time series to generate a single-ROI time-course. All pixels outside astrocytic and vascular ROIs and neuronal cell bodies were specified as neuropil. Vessel diameters were measured using continuous line scans across the vessel that form a space-time image when stacked sequentially. Diameters are extracted from profile changes resulting from expansion or contraction of the intravascular lumen, which is labeled with FITC- or Alexa Fluor 680-dextran.

### Spectral imaging of blood oxygenation

Spectral imaging of blood oxygenation was performed as previously described in^49^. Briefly, six different bandpass filters were placed on a six-position filter wheel (Thorlabs), which was mounted on a DC motor. The center wavelength of the filters ranged from 560 to 610 nm with 10 nm intervals. Light from a tungsten-halogen light source (Oriel, Spectra-Physics) was directed through the filter wheel, which was coupled to a 12 mm fiber bundle. Images were acquired with a cooled 16 bit CCD camera (Cascade 512B, Photometrics). Image acquisition was triggered at ~ 18 Hz by individual filters in the filter wheel passing through an optic sensor. The image set at each wavelength was averaged across trials and averaged data was converted to changes in HbO and Hb at each pixel using the modified Beer Lambert relationship as detailed in ref. ^49^. Baseline concentrations of 60 and 40 μM were assumed for HbO and Hb, respectively^50,51^.

### Two-photon imaging of fluorescent polystyrene beads

The graphene microarray device was fixed at its connecting wire to a 22 × 22 mm glass coverslip with UV-curable optical adhesive (Norland 61). Four 3 mm coverslips serving as spacers were fixed at each corner of the coverslip. One micrometer fluorescent polystyrene beads (Fluoresbrite YG Microspheres, Polysciences, 18860) were diluted to a final dilution of 1:10,000 in ProLong Gold mounting medium (Thermo Fisher, refractive index: 1.47 after curing). A drop of bead-containing mounting medium was added onto a glass slide and the coverslip with array was placed on top. Two-photon imaging was performed after curing of the mounting medium at an excitation wavelength of 800 nm using a 20× UMPlanFI (NA = 0.5) water-immersion objective. Data were analyzed in ImageJ using the MetroloJ plugin^52^.

### Statistics

Improved array performance in vitro has been shown in four independent manufacturing processes with three arrays in each batch.

Animal experiments were designed as proof-of-principle experiments to demonstrate graphene microarray performance in vivo; statistical comparison of experimental groups is therefore not applicable. Structural imaging in GAD67-GFP was performed in two independent experiments; calcium imaging with OGB was performed in four independent experiments; experiments involving OG stimulation were performed in three Thy1-ChR2 animals and one wild-type animal; and macroscopic hemodynamic imaging was performed in one animal.

### Data availability

The data that support the findings of this study are available from the corresponding authors upon reasonable request.

## Electronic supplementary material

Supplementary Information

Description of Additional Supplementary Files

Supplementary Movie 1

Supplementary Movie 2

Supplementary Movie 3
