# Supplementary material for: Deep 2-photon imaging and artifact-free optogenetics through transparent graphene microelectrode arrays
Source: Nat Commun. 2018 May 23;9:2035. doi: 10.1038/s41467-018-04457-5 (PMC5964174; doi:10.1038/s41467-018-04457-5)
Supplement: Supplementary file 1 — Supplementary Information [file 41467_2018_4457_MOESM1_ESM.pdf]

SUPPLEMENT

Thunemann, Lu, et. al.: Deep 2-photon Imaging and Artifact-free Optogenetics through Transparent Graphene Microelectrode Arrays

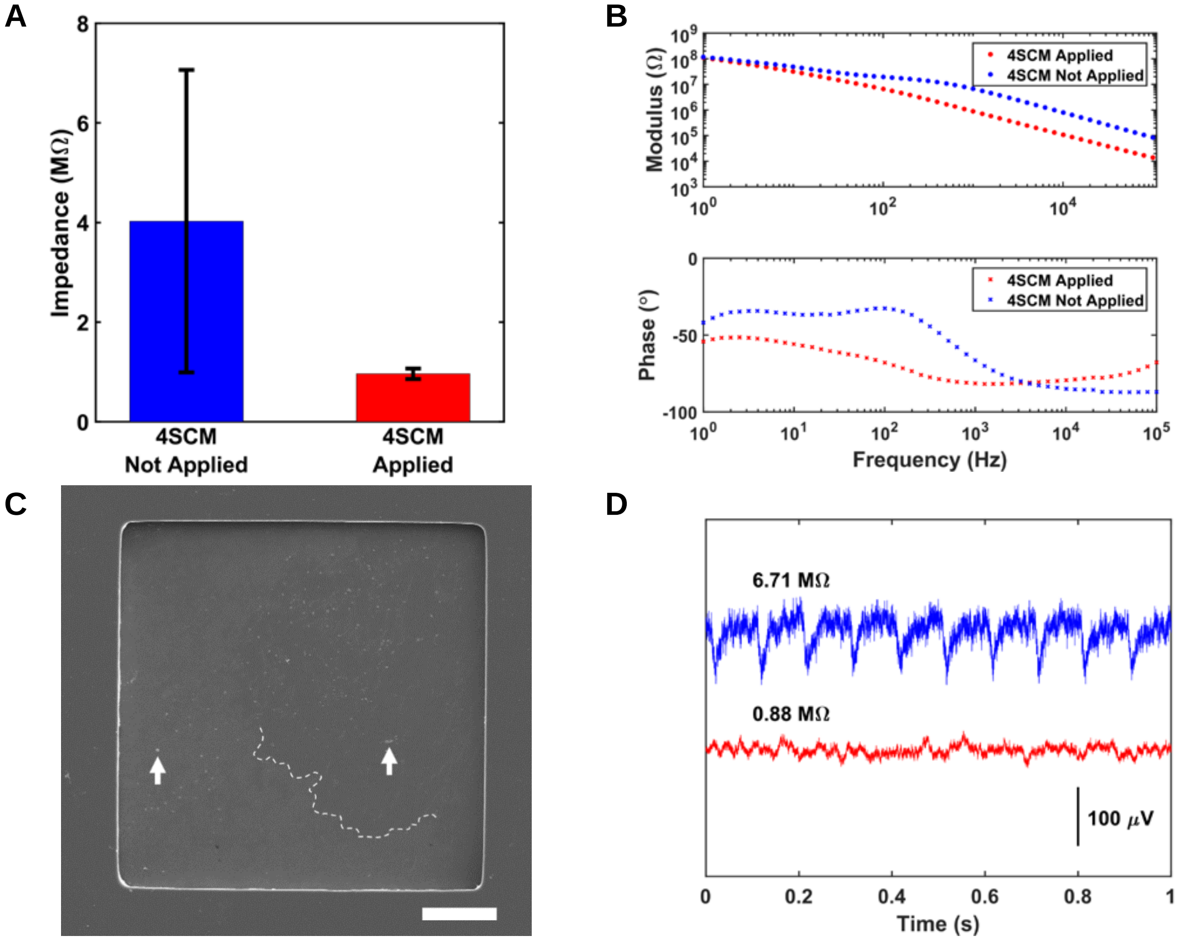

Supplementary Figure 1. The 4-step cleaning method (4SCM) reduces impedance and light-induced artifacts **A.** The 4SCM decreases average impedance (average from 16 channels on one array) from 4.03 MΩ to 963 kΩ. Error bars represent standard deviation of 16 channels from 3 arrays on one wafer. **B.** Electrochemical impedance spectroscopy of one representative channel from an array cleaned with the 4SCM and from one graphene electrode where 4SCM was not applied. **C.** Scanning electron microscopy image of a graphene electrode without 4SCM applied. Residue covers a large portion of the surface of the graphene electrode; white arrows indicate large pieces of residue, the white dashed line indicates the boundary between residue-covered graphene (left, lighter) and clean graphene (right, darker). Scale bar, 20 µm. **D**. Illumination-induced artifacts (10-ms light pulses from a fiber-coupled 470-nm LED at 10 Hz, see also Supplementary Figures 6 and 7) are readily visible when a high-impedance graphene electrode (blue curve, 6.71 MΩ) is illuminated, while no observable light-induced artifacts were recorded upon illumination of a low-impedance graphene electrode (red curve, 0.88 MΩ) on the same array.

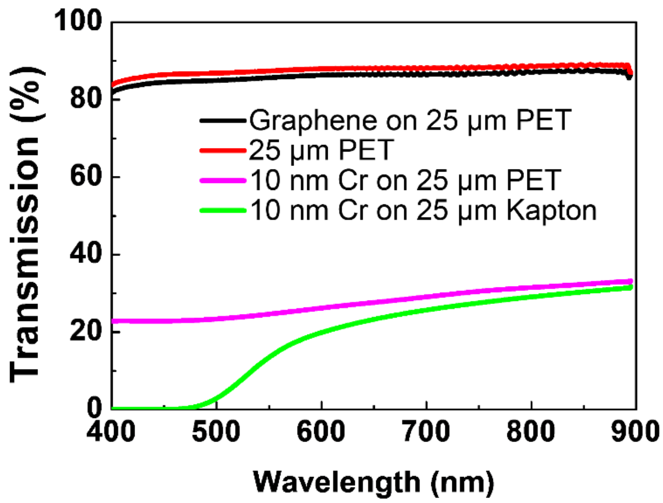

Supplementary Figure 2. Transmission spectrum of graphene on polyethylene terephthalate (PET) compared to 10 nm Cr on PET or Kapton.

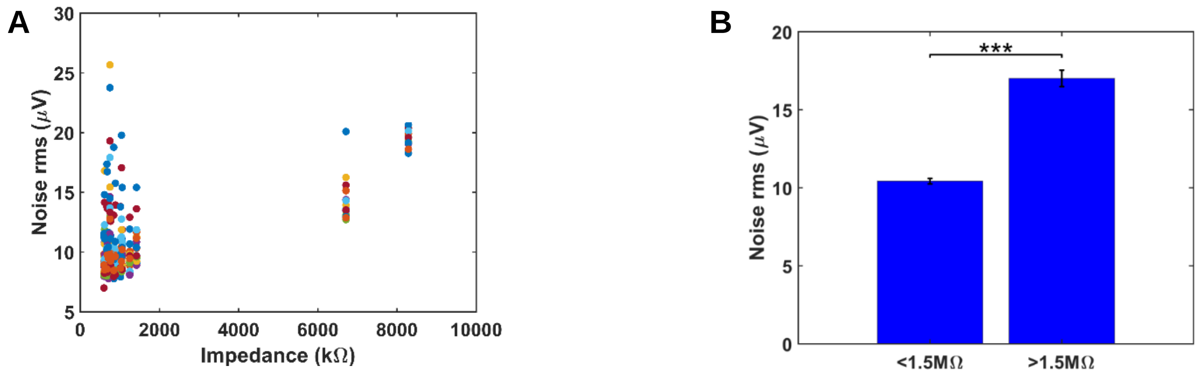

Supplementary Figure 3. Relationship between electrode impedance and noise levels. **A.** Noise levels were estimated for all electrode channels from 16 recording sessions, i.e. 16 data points for each impedance. The root mean square (rms) values of the noise were calculated from high-pass filtered (2 Hz) neural recordings. **B.** Noise level for high-impedance channels (>1.5 MΩ) is significantly higher than for low-impedance channels (<1.5 MΩ). Error bars represent standard error of the mean; *** indicate $p=5.9\times10^{-20}$ (rank sum test in MATLAB).

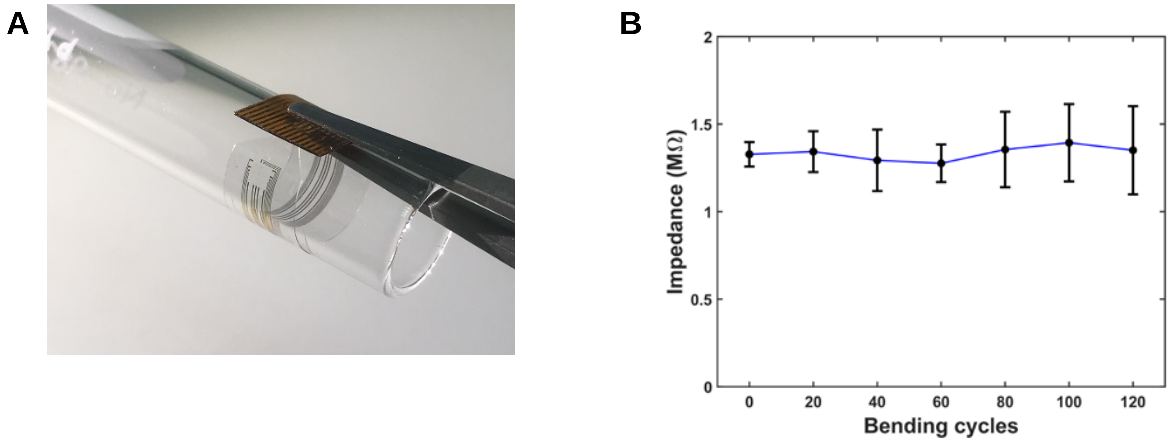

Supplementary Figure 4. Bending test of the graphene microelectrode array. **A.** The array is wrapped around a test tube (radius: 5 mm) to demonstrate the flexibility of the array. **B.** Impedance at 1 kHz remains stable for at least 120 bending cycles (mean ± standard deviation of 3 measured channels in the same array are shown). During one cycle, the array is bent manually to a radius of 5 mm around the test tube (as shown in panel A) and released. Impedance measurements were performed after 20 consecutive bending cycles.

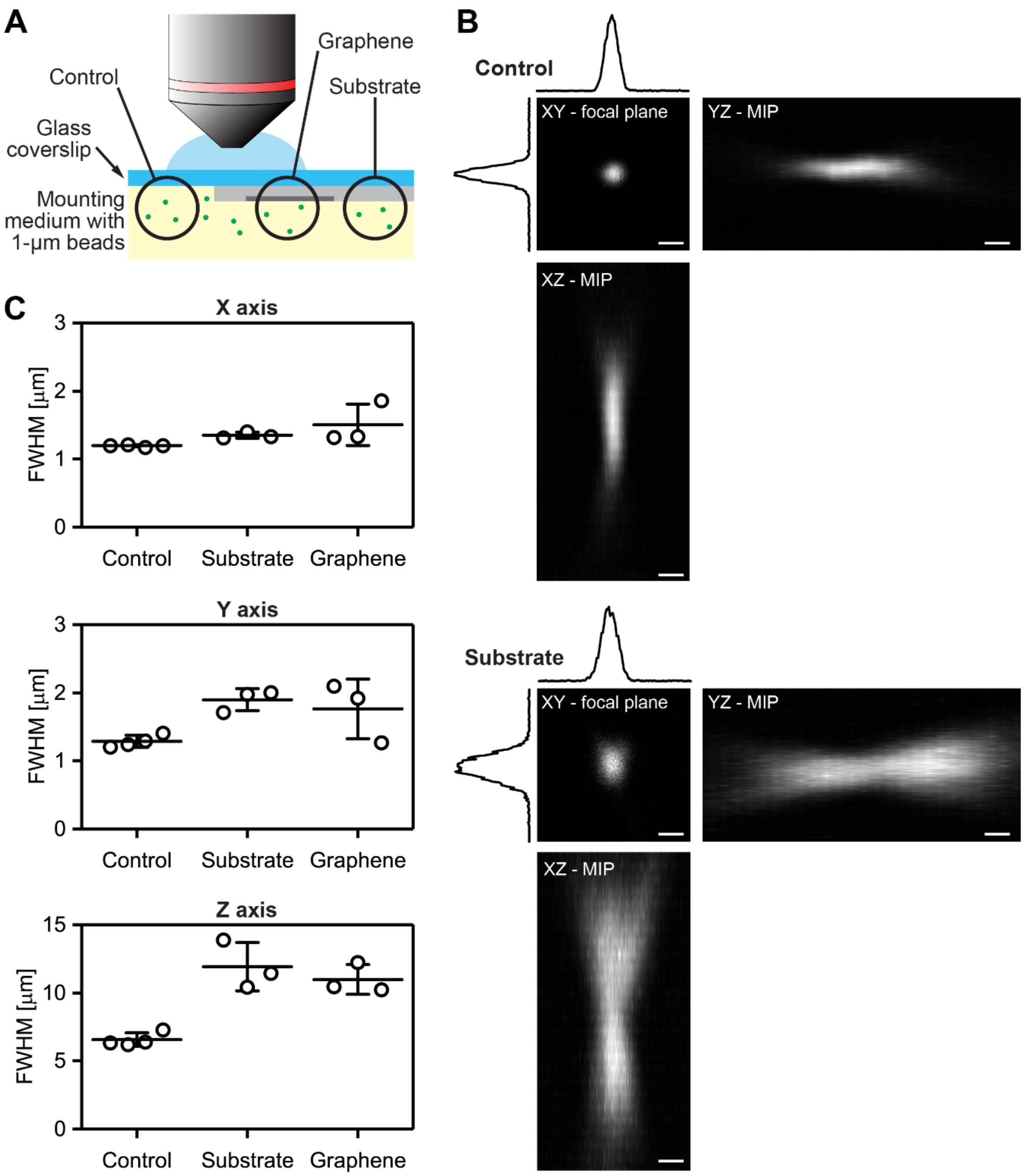

Supplementary Figure 5. *In vitro* test of 2-photon imaging resolution with fluorescent beads. **A.** 1-µm fluorescent beads were embedded in mounting medium. Z stacks (200×200 pixels, pixel size: 100×100 nm, Z step size: 1 µm) of individual beads beside the array (Control), below the array substrate (Substrate), and below graphene microelectrode (Graphene) were acquired with 2-photon excitation at 800 nm through a 20×/0.5 NA objective. **B.** Representative intensity profiles (after normalization to maximum intensity within the image) of beads beside the array (Control) and below the array (Substrate). The XY image shows the focal plane with respective line profiles along X and Y axis; XZ and YZ images are maximum intensity projections (MIPs) along the third axis. Scale bars, 1 µm **C.** Quantification of full width at half-maximum (FWHM) with a Gaussian peak function (performed in MetroloJ plugin in ImageJ). Mean ± standard deviation of 3-4 beads per condition are shown (symbols represent average of 3 measurements of an individual bead).

A 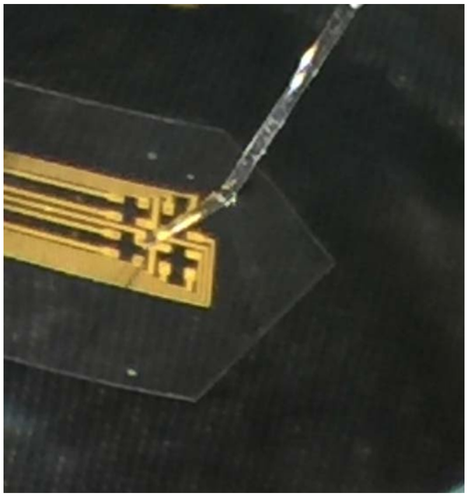 B 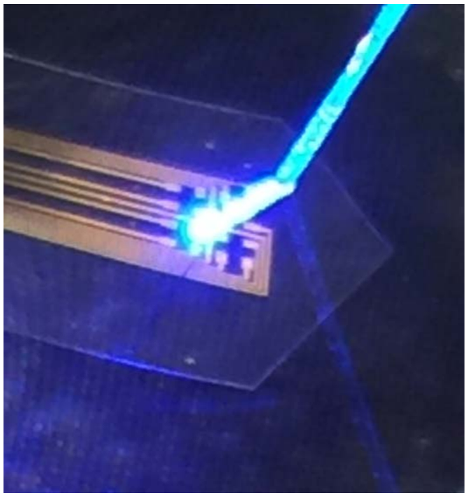

Supplementary Figure 6. Setup for in-vitro characterization of light-induced artifacts; shown here is a gold electrode array with (A) 470-nm LED stimulation off and (B) 470-nm LED stimulation on.

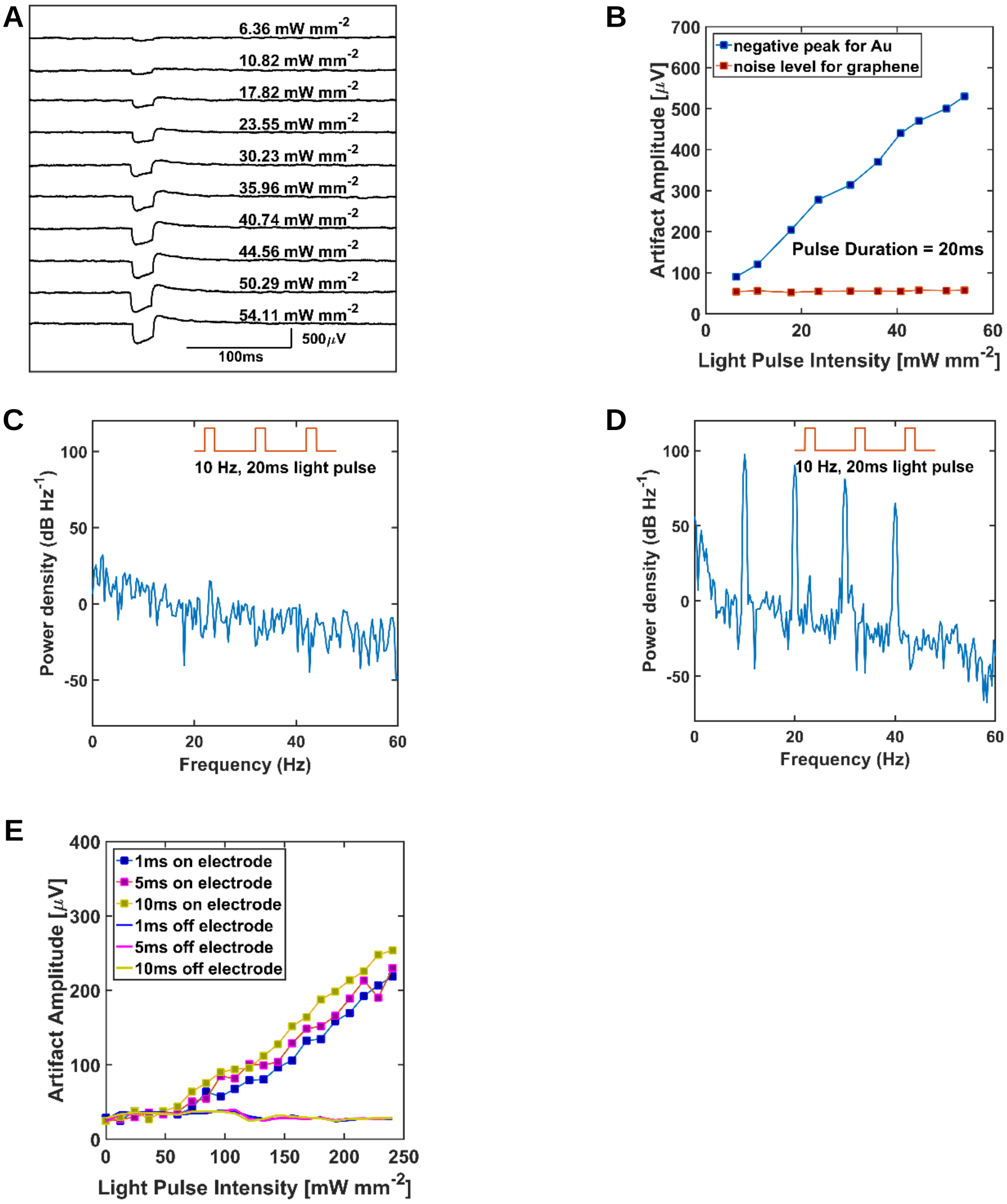

Supplementary Figure 7. *In vitro* analysis of light-induced artifacts in graphene and gold microelectrode arrays. **A.** Time series of light-induced artifacts in gold microelectrode arrays upon illumination with 0-54.1 mW mm^{-2} (20-ms light pulse from a fiber-coupled 470-nm LED). Measurements were performed in PBS **B.** Artifact amplitudes for graphene and gold electrode in dependence of laser pulse intensity. **C, D**. Power spectra for 10-Hz illumination of graphene (C) or gold electrode (D). **E**. Amplitudes of light-induced artifacts for a graphene electrode upon illumination with 473-nm laser light with 0-240.7 mW mm^{2} for 1, 5, or 10 ms through a 20× microscope objective (using the same setup as for animal experiments shown in Figure 5 and 6). Amplitude of the light-induced artifact of the graphene electrode is below the noise level for light pulse intensities <60.2 mW mm^{-2}. In this setup, the graphene microelectrode array was placed on an agarose phantom and covered with a glass coverslip.

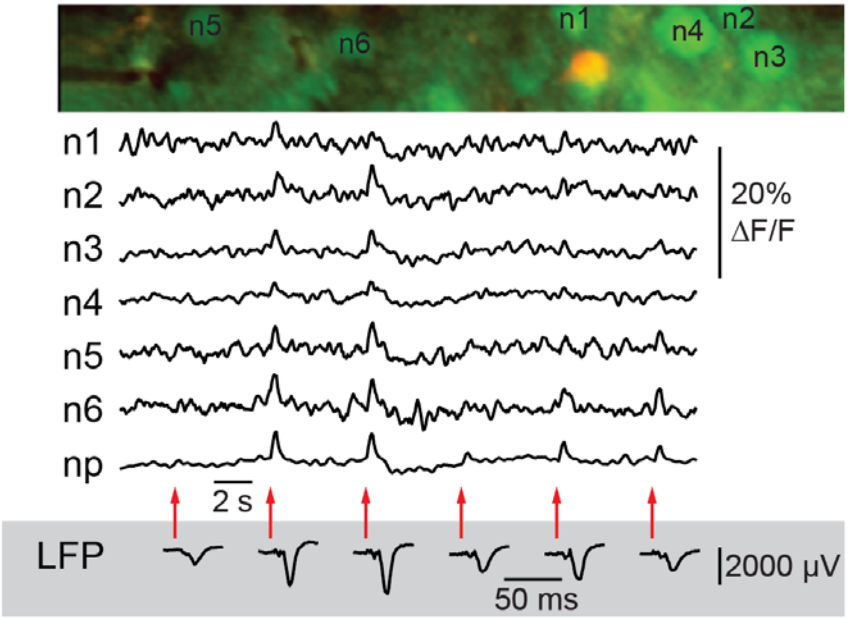

Supplementary Figure 8. Additional experiment for correlation of Ca^{2+} signal and LFP amplitude. A representative field of view (FOV, top) containing six OGB1-stained neurons (green) and one SR101/OGB1-stained astrocyte (yellow), respective Ca^{2+} traces (middle) of neurons (n1-n6) and neuropil (np) given as ΔF/F, and corresponding LFP recordings (bottom) of the graphene microelectrode adjacent to the FOV used for Ca^{2+} imaging (6 trials in one run are shown). Red arrows indicate delivery of a single electric stimulus (300 μs, 1 mA) to the contralateral whisker pad.

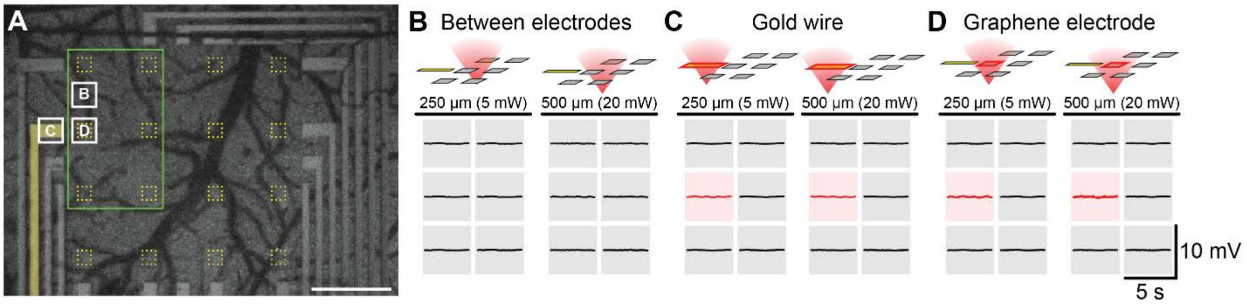

Supplementary Figure 9. Two-photon laser scanning induces only minor artifacts in electrical recordings with graphene electrodes in vivo. **A**. Overview of the preparation with graphene microelectrode array. Yellow outlines represent single graphene electrodes, white rectangles indicate locations of 2-photon imaging, the green rectangle indicates which electrode traces are shown in panels B-D. Scale bar, 500 µm. **B.** Imaging was performed between two graphene electrodes. **C.** The 2-photon laser beam was focused on a gold wire (highlighted in yellow in panel A); the respective electrode channel is highlighted in red. **D.** The 2-photon laser beam is focused on a graphene electrode; the corresponding electrode channel is highlighted in red. In B-D, recordings from 6 electrodes (corresponding to the green rectangle in panel A) are shown. For each location, imaging was performed at a depth of 250 µm below the surface with a laser power of 5 mW and at a depth of 500 µm with a power of 20 mW. The laser was set to an excitation wavelength of 800 nm; imaging was performed with a 20× objective and 4× optical zoom to acquire images with a size of 150 µm×150 µm at a resolution of 512×512 pixels.

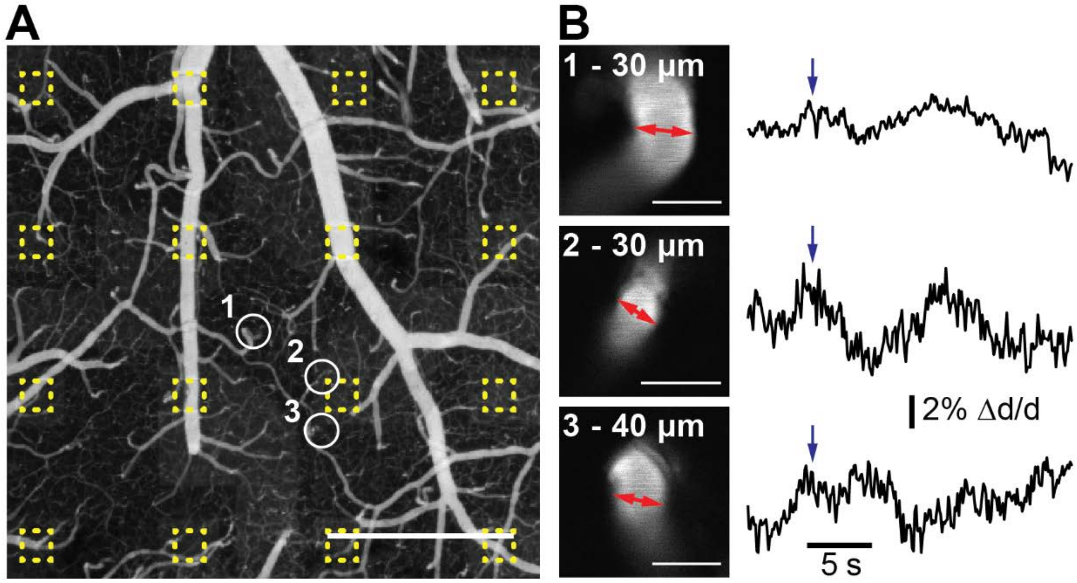

Supplementary Figure 10. Vascular responses are absent upon 473-nm laser illumination in a wild-type (ChR2-negative) animal. **A.** As in experiments with Thy1-ChR2 mice (see **Figure 6**), blood plasma was labeled with 2-MDa FITC-dextran, and single cortical arterioles (at locations 1, 2, and 3) were imaged in line-scan mode. Yellow squares indicate positions of individual graphene electrodes. Scale bar, 500 µm. **B.** Reference images of the 3 arterioles (left) and corresponding time courses of vessel diameters changes (right, 3 trials per arteriole). Red arrows indicate where line scans were performed, blue arrows indicate illumination with 473-nm laser through the objective (100 ms, 7.1 mW). Scale bars, 20 µm.
