# Supplementary material for: Deep 2-photon imaging and artifact-free optogenetics through transparent graphene microelectrode arrays
Source: Nat Commun. 2018 May 23;9:2035. doi: 10.1038/s41467-018-04457-5 (PMC5964174; doi:10.1038/s41467-018-04457-5)
Supplement: Supplementary file 2 — Description of Additional Supplementary Files [file 41467_2018_4457_MOESM2_ESM.pdf]

Description of Additional Supplementary Files

File Name: Supplementary Movie 1
Description: Spatiotemporal dynamics of the electrical response to the stimuli (6 trials with 1 stimulus per trial) as shown in **Figure 3E**. Electrical recordings at 1 kHz of all 16 channels are shown in overlay with the microscopic image from **Figure 3A**; for every of the 6 trials; a period of 50 ms is shown, covering 10 ms before until 40 ms after stimulus delivery. Note that in trials 3-5, no or very small responses are present.

File Name: Supplementary Movie 2
Description: Spatiotemporal dynamics of the hemodynamic response (average of 10 trials) to the stimulus as shown in **Figure 7C** and **F-H**. The video shows relative changes of HbO, Hb, and HbT over a period of 9.2 s, starting 1 s before onset of stimulus delivery.

File Name: Supplementary Movie 3
Description: Spatiotemporal dynamics of the electrical response (average of 10 trials) to the six stimuli as shown in **Figure 7D** and **E**. Electrical recordings at 1 kHz of all 16 channels are shown in overlay with the image from **Figure 7A**; for every of the 6 stimuli, a period of 50 ms is shown, covering 10 ms before until 40 ms after stimulus delivery.
